# A Common Language: How Neuroimmunological Cross Talk Regulates Adult Hippocampal Neurogenesis

**DOI:** 10.1155/2016/1681590

**Published:** 2016-04-10

**Authors:** Odette Leiter, Gerd Kempermann, Tara L. Walker

**Affiliations:** ^1^Center for Regenerative Therapies Dresden (CRTD), Technische Universitaet Dresden, 01307 Dresden, Germany; ^2^German Center for Neurodegenerative Diseases (DZNE) Dresden, 01307 Dresden, Germany

## Abstract

Immune regulation of the brain is generally studied in the context of injury or disease. Less is known about how the immune system regulates the brain during normal brain function. Recent work has redefined the field of neuroimmunology and, as long as their recruitment and activation are well regulated, immune cells are now known to have protective properties within the central nervous system in maintaining brain health. Adult neurogenesis, the process of new neuron generation in the adult brain, is highly plastic and regulated by diverse extrinsic and intrinsic cues. Emerging research has shown that immune cells and their secreted factors can influence adult neurogenesis, both under baseline conditions and during conditions known to change neurogenesis levels, such as aging and learning in an enriched environment. This review will discuss how, under nonpathological conditions, the immune system can interact with the neural stem cells to regulate adult neurogenesis with particular focus on the hippocampus—a region crucial for learning and memory.

## 1. Introduction

Adult neurogenesis occurs predominantly in two specialized stem cell niches, the subgranular zone (SGZ) of the dentate gyrus and the subventricular zone (SVZ) below the ependymal lining of the lateral ventricles. In these neurogenic niches precursor cells reside in close proximity to the microvasculature, indicating a close connection between the nervous system and the circulation. In the hippocampus, the multistep process of neurogenesis is regulated on different levels by intrinsic and extrinsic factors that have either a positive or negative influence, or even both. Physical exercise and environmental enrichment, mainly increasing precursor cell proliferation and survival of the newborn cells, respectively, represent two of the most prominent positive external neurogenic stimuli [1–3]. Aging and stress on the other hand lead to a reduction in hippocampal neurogenesis [4, 5]. All of the above examples are linked to changes in the physiological environment and recent data, especially in the field of aging, have revealed how changes in the circulatory system can influence the brain [6–8].

The brain is generally considered an immunologically privileged organ and it has long been assumed that cross talk between the immune and nervous systems is unlikely due to their physical separation by the blood brain barrier (BBB). It seemed that the brain functioned optimally when no immune cells were present. Work over the past decade, however, has made major advances in the field of neuroimmunology. The recent rediscovery of the central nervous system (CNS) lymphatic system that drains immune cells from the cerebral spinal fluid (CSF) to the lymph nodes has further highlighted this route of communication [9, 10]. The brain can now be considered to some extent to have immune properties, as it is able to respond to injury, degeneration, or infection, albeit in a different way than the peripheral immune system. We are only beginning to understand the importance of the immune system in maintaining brain homeostasis under nonpathological conditions and the exact mechanisms of how neuroimmunological communication maintains the tight regulatory balance required for proper brain function have yet to be fully elucidated. In this review we will explore the close association between the hippocampal stem cell niche and the microvasculature. We will outline the multitude of both brain resident and peripheral immune cells and discuss the potential significance of their cross talk with the neural stem cells, an interaction that is facilitated by a plethora of secreted immune molecules. In the context of aging, we will describe how the circulatory system can influence the brain and raise the interesting question of whether blood cells could provide a source of new neurons.

## 2. The Hippocampal Microvasculature Is a Gateway between the Circulatory System and the Nervous System

The BBB is formed by tight junctions between endothelial cells and is ensheathed by the endfeet of perivascular astrocytes and pericytes. Due to their physical separation by the BBB, cross talk between the immune and nervous systems was generally considered unlikely. However, despite its name, the BBB is dynamic and allows, when required, peripheral immune cells to enter the brain under both physiological and pathological conditions [11, 12]. Circulating immune cells and blood-borne factors travelling through the bloodstream into the brain might be more involved in the regulation of neurogenesis than long thought. The finding that nestin-GFP-expressing hippocampal precursor cells directly contact the niche-resident endothelial cells via vascular endfeet [13, 14] supports this hypothesis.

The hippocampus is a highly vascularized area in the brain and proliferating adult hippocampal precursor cells reside in close proximity to blood vessels and dividing endothelial cells [15]. This suggests that the hippocampal stem cell niche, although located deeply inside the brain, receives extrinsic cues via circulating and endothelium-derived factors [15]. Factors secreted by endothelial cells affect proliferation, differentiation, and survival of neural stem and progenitor cells and include brain derived neurotrophic factor (BDNF) [14, 16, 17] and vascular endothelial growth factor (VEGF) [15, 18, 19]. VEGF stimulates precursor cell proliferation in the SGZ and has neurogenic and angiogenic properties, indicating a direct link between the blood system and the brain [15, 19]. Following exercise, a strong physiological stimulus of hippocampal neurogenesis [2, 3], raised levels of circulating VEGF mediate the physical activity-induced increase in new neuron production [20]. Interestingly, the blockade of peripheral VEGF prevents the run-induced increase in precursor proliferation but does not affect basal neurogenesis in nonrunning animals, suggesting that peripheral factors can affect neurogenesis by different, independent mechanisms [20].

Despite their importance in maintaining a functional niche environment, the vascular niches within the two major neurogenic regions differ from each other. Unlike the hippocampus, where neurogenesis and angiogenesis appear to be coupled, no dividing endothelial cells are observed in the SVZ [15, 21]. The vascular difference between the niches could be of importance for stem cell regulation by external stimuli, such as environmental enrichment and physical exercise, that affect neurogenesis in the hippocampus but not in the SVZ [22]. In this context, signals from the CSF that repress proliferation in the SVZ cannot be excluded, as SVZ stem cells contact both blood vessels and the CSF [23–26].

The proximity of the niche to the blood vessels allows the supply of nutrients or signals to maintain homeostasis under physiological conditions, and vascular changes occur during normal aging and in the context of neurodegenerative diseases [27, 28]. The aged vascular system in the brain is characterized by decreased blood flow and the functional decline of the BBB carrier system [27], indicating an altered supply of circulating factors from the blood stream, possibly leading to either the enhanced or decreased availability of signaling molecules.

## 3. Brain Resident Immune Cells

**Figure 1:**
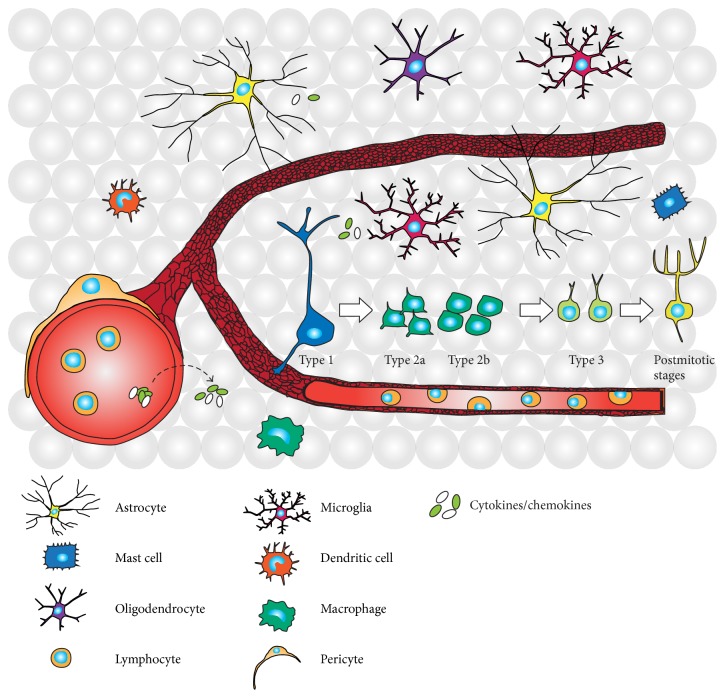
Neural progenitor cells and immune cells coexist in the hippocampal neurogenic niche. Type 1 neural stem cells in the subgranular zone of the hippocampal dentate gyrus mature through different developmental stages during the multistep process of new neuron formation. Type 1 radial glia-like cells give rise to transiently amplifying progenitor cells (Type 2a/Type 2b). After passing the neuronally committed Type 3 stage the cells become postmitotic after which they integrate as mature granule cells into the existing hippocampal circuitry. Within the complex niche environment peripheral and resident immune cells interact with niche cells to regulate the neurogenic process under physiological conditions. In addition, immune cells in the blood and the niche secrete immune molecules, including cytokines and chemokines, to facilitate neuroimmunological communication.

The hippocampal neurogenic niche is complex and many types of cells interact to maintain a functional niche environment (Figure 1). Apart from the neural stem and neuronal lineage cells, many niche-resident cells have immunological characteristics that, in the steady state, can regulate adult neurogenesis. The largest populations are the niche-resident microglia and astrocytes, which have immune properties and are important for local surveillance [29–31].

### 3.1. Microglia

Microglia, although considered to be a type of immune cell, are an integral part of the brain. These resident macrophages are uniformly arranged throughout the brain and constitute approximately 10–15% of total brain cells. Microglia originate from macrophages during hematopoiesis in the yolk sac and migrate to the neural tube where they form microglia. Because bone marrow monocytes do not contribute to the pool of mature microglia in a healthy brain, it is suggested that they are sustained by local self-renewing progenitor cells [32].

Microglia located in the neurogenic niches are interesting candidates for the regulation of adult neurogenesis, both in baseline and in injury states. Under baseline conditions microglia are relatively quiescent. These resting microglia have a ramified morphology with many processes, which they use to survey the surrounding area for damage or infection [33]. Under resting conditions microglia perform important functions during development including phagocytosis of debris resulting from cell apoptosis and promote neuronal apoptosis [34] and mice deficient in microglia have severe postnatal brain development defects [35]. Microglia also play a key role in the active remodeling of the presynaptic environment by engulfing presynaptic termini [36]. This synaptic pruning is dependent on the complement system [37], whereby C1q produced by neurons activates a cascade that ultimately activates the microglial expressed C3R and these microglia preferentially engulf inactive synapses [38]. Another signaling pathway is via the neural cell secreted cytokine CX3CL1 and its corresponding microglial based receptor CX3CR1. CX3CR1 deficient knockout mice have adult neurogenesis deficits [39], impaired learning and memory, and an inability to achieve long-term potentiation (LTP) in the hippocampus [40].

Following an insult or infection microglia respond to signals from the peripheral immune system to induce neuroinflammation. Following activation, microglia change their shape becoming amoeboid and phagocytose cell debris. They also release factors such as proteases, neurotrophic factors, cytokines, and reactive oxygen species (ROS). Microglia responses are heterogeneous and result in different activation phenotypes which are broadly classified into the classical M1 and the alternative M2 states [41]. Classical activation occurs when the microglia detect a foreign antigen and act as the first line of defense by transitioning from innate immunity into an adaptive immune response by recruiting peripheral immune cells. The most common method to experimentally induce classical microglial activation is via administration of the bacterial endotoxin lipopolysaccharide (LPS) [42]. Such work demonstrated that classically activated microglia impair adult hippocampal neurogenesis without affecting proliferation—an effect that could be blocked with the microglial inhibitor minocycline [42, 43]. Classically activated or proinflammatory M1 microglia were associated with reduced neurogenesis and this effect is believed to be due to their production of nitric oxide [44, 45] and ROS [46], whereas neuroprotective M2 microglia stimulate neurogenesis via the release of anti-inflammatory cytokines and growth factors. The neuroprotective M2 response occurs when switching from the classical inflammatory response to a reduction of proinflammatory factors towards a production of neuroprotective factors involved in repair. This switch is induced by stimulation with anti-inflammatory cytokines such as IL-4 or IL-13 resulting in the downregulation of classical activation genes and the upregulation of repair genes [47]. Alternatively activated M2 microglia have an increased expression of a number of anti-inflammatory cytokines including interleukin-10 (IL-10), transforming growth factor beta (TGF-*β*), and growth factors such as insulin-like growth factor (IGF), nerve growth factor (NGF), and BDNF. It has been suggested that M2 microglia might be important for the maintenance of neurogenesis, as a number of the anti-inflammatory cytokines produced have been found to support adult neurogenesis [48].

Microglia are controlled by both intrinsic and extrinsic systems. They can be modulated by neuronal activity via neurotransmitters, as well as directly influencing neuronal activity and other regulatory molecules. They can also be regulated by astrocytes and release soluble factors in the adult brain [49]. Microglia can also upregulate major histocompatibility complex class II (MHC II), an important regulator of the cellular immune response that is responsible for the presentation of antigens to CD4^+^ T cells [50].

Physical activity, a well-known stimulus of adult hippocampal neurogenesis, increases proliferation of microglia in both the cortex and hippocampus [51]. It has been supported by* in vitro* experiments that the exercise induced increase in hippocampal precursor proliferation is mediated via microglia and can be abolished when microglia are removed from the cultures. The potential mediator of this effect is signaling along the CX3CL1-CX3CR1 axis [52]. In addition, it was shown that microglia from exercising mice were able to activate the latent precursor cells from sedentary mice* in vitro* [52]. Conversely, microglia might also play a role in the age-related decline in neurogenesis [53]. Aging is associated with increased microglia activation, particularly towards a classic activation phenotype, with increased basal levels of the proinflammatory cytokines tumor necrosis factor alpha (TNF-*α*), interleukin 6 (IL-6), and interleukin-1 beta (IL-1*β*) [53].

### 3.2. Astrocytes

Astrocytes, the most abundant cell type in the brain, are located throughout the CNS. In the healthy CNS astrocytes have many important functions including providing metabolic support [54], water homeostasis [55], exchanging information between neurons and blood vessels to coordinate oxygen and glucose delivery, control of extracellular ion flux and antioxidant support by glutathione release [56], directing the development of synapses [57], modulating cerebral blood flow [58], the uptake and clearance of neurotransmitters such as GABA and glutamate [59], and helping to maintain the integrity of the BBB [60].

Astrocytes also play an important role in the brain's defense mechanism and they have phagocytic and antigen-presenting capacity [29–31]. Astrocytes can express major histocompatibility complex class I (MHC I) and MHC II antigens when stimulated by interferon gamma (IFN-*γ*)* in vitro*, which can lead to T cell activation [61]. Whether astrocytes express MHC II* in vivo* is controversial and may be limited to pathological conditions such as multiple sclerosis [29]. Astrocytes are also key players in the CNS as the targets and effectors of many cytokines and other inflammatory molecules. Reactive astrocytes, produced in immediate response to CNS trauma, form the glial scar and are thought to be beneficial by preventing contact between the damaged and healthy neurons. Following trauma or infection reactive astrocytes can produce a number of cytokines (e.g., CXCL10, CCL2, and IL-6) that mediate innate immune function such as recruitment of monocytes and microglia [61]. Astrocytes can also respond to a number of cytokines (TNF-*α*, EGF, FGF, and a number of interleukins) themselves and display a number of receptors involved in innate immunity including toll-like receptors (TLRs) [62, 63]. In contrast to the immediate activation seen following trauma, activation of astrocytes that occurs later inhibits regeneration and contributes to sustained inflammation [64]. Reactive astrocytes are key players in neurodegenerative diseases such as amyotrophic lateral sclerosis and Alzheimer's disease. The strongest evidence for a role of astrocytes in CNS autoimmune disease is in neuromyelitis optica, an inflammatory disease that besides its occurrence in the context of multiple sclerosis can also be caused by specific antibodies to the water channel protein Aquaporin-4, which in the CNS is located exclusively on the surface of astrocytes [65]. It was also shown that in some multiple sclerosis patients there are autoantibodies against the potassium channel Kir4.1 that is also present on astrocytes [66].

Apart from the obvious role of a subset of astrocytes that function as the neural stem cells (NSC) under homeostatic conditions, astrocytes can also release soluble factors which influence distinct stages of adult neurogenesis including stem cell proliferation [67], neuronal differentiation [68], neuronal survival [69], and synaptic integration [70]. CNS injuries can also produce reactive astrocytes with NSC potential [71]. This is in contrast to those astrocytes activated during neurodegeneration that do not have stem cell potential [71]. Conversely, blocking reactive gliosis leads to improved integration of transplanted neural precursor cells (NPCs) in the mouse hippocampus [72].

### 3.3. Pericytes

Pericytes are smooth muscle-derived cells that are important in maintaining the BBB. Pericytes are often referred to as the macrophages of the brain, as due to their MHC II dependent antigen-presenting ability and their direct contact with the microvasculature they represent one of the first lines of defense [73]. In response to infection, pericytes mount an inflammatory response by increasing expression of proinflammatory cytokines, such as IL-6, TNF-*α*, and IL-1*β* [74, 75]. There are several reports that pericytes have neurogenic potential being able to differentiate into neurons* in vitro* and* in vivo* [76–78] and can be converted into neurons by direct reprogramming [79]. Recently, diffusible factors produced by pericytes were shown to increase NSC proliferation and neurogenesis* in vitro* [80].

### 3.4. Perivascular Macrophages

Perivascular macrophages are myeloid cells that are continuously replenished from the bone marrow progenitor cells and are located within the perivascular space. They wrap their processes around the vasculature and help the BBB formation [81]. The major roles of perivascular macrophages include phagocytosis of cellular and pathogenic debris, brain surveillance of the interstitial space via pinocytosis, and initiation of the CNS acute phase response via the production of prostaglandins [82]. Interestingly it was shown that the percentage of TLR4^+^/CD14^+^ brain macrophages increased when mice were housed in an enriched environment potentially allowing leukocytes in the microvessels to release various neuroprotective and anti-inflammatory factors [83]. Perivascular macrophages also play a role in neurodegeneration [84], although whether they are involved in the regulation of adult neurogenesis under nonpathological conditions remains unclear.

## 4. Peripheral Immune Cells Influence Adult Neurogenesis

In addition to the brain resident immune cells, peripheral immune cells including T cells, B cells, natural killer cells, macrophages, mast cells, and dendritic cells can enter the CNS under normal conditions.

### 4.1. T Cells

Peripheral T cells can selectively enter the brain through the choroid plexus, a barrier that is composed of epithelial tight junctions and fenestrated epithelial cells, when required [85, 86]; however under physiological conditions they are present in very low numbers [87]. T cells can hone to sites of damage in the brain, become activated, and activate resident microglia, in a way that is different to the classical inflammation activation of microglia, to make them supportive of neuronal survival and proliferation. Interestingly, the CD4^+^ T cells within the choroid plexus are distinct from those in the circulating blood as they express T cell receptors specific for CNS antigens, and they are of effector-memory type in contrast to those of the CSF which are central-memory T cells [88].

The first evidence of a role of T cells in adult neurogenesis was from Butovsky and colleagues in 2006 [89] who demonstrated that microglia activated by T helper (Th) cells produced the cytokines interleukin-4 (IL-4) and IFN-*γ* that promoted neurogenesis* in vitro*. Th1 and Th2 cells are generally thought to be detrimental to neurogenesis via the release of IFN-*γ*, although they can also be neuroprotective via the action of their main anti-inflammatory cytokine, IL-4 [90].

This evidence was closely followed by an* in vivo* corroboration of this link by Ziv and colleagues (2006) [91]. They showed that CD4^+^ T cells promote and maintain neurogenesis by activating microglia via the release of soluble cytokines and regulating IGF-1 transport into the brain, thus regulating BDNF levels [91]. They also demonstrated that adult neurogenesis was impaired in immune-deficient severe combined immunodeficiency (SCID) and nude mice but could be restored by injection of T cells recognizing an antigen specific for the central nervous system [91]. Interestingly, CD4^+^ T cells have been identified as the key players in this process. A study from our lab found that repopulation with CD4^+^ but not CD8^+^ T cells was able to restore the decrease in adult hippocampal neurogenesis observed in immune-deficient mice [92], thereby highlighting CD4^+^ T cells as the proneurogenic T cell population under physiological conditions. An increase in activated CD4^+^ T cells could be observed in the meninges of mice after training in the Morris water maze [93] indicating an impact of brain activity (learning) on the immune system. In these experiments, CD4^+^ T cells displayed an activated phenotype and produced increased levels of IL-4. In addition, SCID mice that are devoid of mature T cells have dramatic impairments in hippocampal-dependent spatial learning and memory tasks including the water maze [94], the Barnes maze [95], the radial arm water maze [95], and the novel object recognition test [96]. Whether T cells also play a role in regulating the proneurogenic response to physical activity, however, remains unknown.

### 4.2. Regulatory T Cells

Regulatory T cells (Tregs) are a subpopulation of T cells, which normally work to suppress autoimmune responses and thus are critically involved in maintaining immune homeostasis. Interestingly, they can also produce neurotrophic factors and activate resident microglia [97]. The extent of this regulatory function is genetically determined and can differ between strains and individuals [98, 99]. Foxp3^+^ Tregs are involved in suppressing the immune response in conditions including Alzheimer's disease, Parkinson's disease, traumatic brain injury, and stroke [100]. Tregs in the brain have mainly been studied following stroke, and their role in neurogenesis under physiological conditions is still unclear. It has been demonstrated that Tregs are present in the normal rat brain in the cortex, subcortical regions, hippocampus, and choroid plexus [97]. Recently it was shown that activated Tregs injected into the lateral ventricle of mice increased NPC proliferation, but nonactivated Tregs had no effect [101]. Activated Tregs stimulated NSC proliferation in the SVZ after middle cerebral artery occlusion but did not improve stroke outcome, as defined by infarct volume. In addition, activated Tregs enhanced the proliferation of passaged neurospheres* in vitro*, an effect that could be blocked with IL-10-specific neutralizing antibodies [101].

### 4.3. B Cells

B cells, the other major class of lymphocytes, can also become activated and enter the healthy human brain, albeit in very low numbers [102]. B cells however can be found in larger numbers in the brains of patients with multiple sclerosis where they play a role in the pathogenesis of the relapsing inflammatory subtype [103]. One study claimed that B cells are not required for normal hippocampal NSC proliferation [104] but the function of B cells in the healthy brain is still unknown. Given the fact that there is growing evidence of B lymphocyte functions beyond combating infection, for example, including roles in autoimmunity, this question will have to be reconsidered in the future.

### 4.4. Natural Killer Cells

Natural killer (NK) cells are part of the innate immune system and have heterogeneous phenotypes and functions. Despite their primary function to kill aberrant cells, NK cells also exert immunoregulatory functions and orchestrate adaptive immune responses through the interaction with antigen-presenting cells, T cells, and B cells [105]. Although they comprise about 10% of lymphoid cells in the healthy brain [106], the role of brain resident NK cells in CNS homeostasis is not clear. Mainly studied in pathology such as in the context of multiple sclerosis, brain NK cells have neuroprotective [107] as well as neurotoxic [108] effects. During chronic brain inflammation, NK cells reside in close proximity to NSCs in the SVZ and the interaction between NK cells and NSCs results in reduced proliferation and decreased numbers of GFAP^+^BrdU^+^ cells [109]. Considering their regulatory function, the extent to which NK cells interact with NSCs during normal brain function or in response to proneurogenic stimuli such as physical exercise or environmental enrichment remains unknown.

### 4.5. Dendritic Cells

Dendritic cells are the antigen-presenting cells of the immune system and their role is to present antigens to T lymphocytes to induce either tolerance or adaptive immune responses. Until recently, the brain was believed to be devoid of dendritic cells under steady-state conditions. However, dendritic cells have now been shown to infiltrate into the brain via the choroid plexus, nasal epithelium, meninges, and blood in response to autoimmunity, injury, aging, and infection [110, 111]. Despite much progress, the complete picture of dendritic cell participation in the brain's immune resonance is far from being completely understood. In addition, the presence of dendritic cells in the steady-state brain of humans is unclear and warrants further investigation.

### 4.6. Mast Cells

Mast cells, most well known for their role in allergy and anaphylaxis, are involved in the histamine response. Mast cells are generated in the bone marrow and under normal conditions can migrate to various areas of the brain including the hippocampus. Mast cell-deficient mice have hippocampal learning deficits and deficits in hippocampal, but not SVZ, neurogenesis [112]. Mast cells are a source of serotonin and treatment of the mast cell-deficient mice with fluoxetine, a selective serotonin reuptake inhibitor, can reverse the deficit in adult neurogenesis [112]. Interestingly, mast cells can be activated by physical activity [113]. Whether these cells play a role in the increase in adult hippocampal neurogenesis in response to physical activity, however, is still unknown.

## 5. Immune Molecules

The various immune cells described above secrete a plethora of immune molecules, including pro- and anti-inflammatory cytokines that play an important role in brain function, both in maintaining the physiological state and in disease conditions. This regulation is tightly controlled and the outcome depends on their concentration, the particular type of cell that is activated, and other factors that are also secreted by other populations of cells in the brain.

### 5.1. Cytokines

Immune and neural cells share a common language for communication—cytokines. Cytokines, the chemical messengers of the immune system, although typically thought of in this context, are also important for normal brain function. In the brain, cytokines are mainly produced by activated microglia as part of the innate immune response. Increased cytokine production in the brain is generally regarded as detrimental, as it is usually associated with conditions of inflammation, infection, and neurodegeneration. The role of cytokines in normal brain function, however, is less well studied [114].

Immune cells produce a multitude of different cytokines. We will briefly outline the best studied of these, IL-6, TNF-*α*, IL-1*β*, and IFN-*γ*, with the major focus on their role in hippocampal neural stem cell regulation. IL-6 can act as both a pro- and anti-inflammatory cytokine and therefore its specific role in hippocampal learning and memory depends on the context. Adult hippocampal neurogenesis is dramatically reduced in the presence of IL-6 and inhibition of IL-6 can restore this deficit [42]. In addition, IL-6 is involved in regulation of synaptic plasticity, LTP, and memory [115] and there is an age dependent increase in IL-6 that is accompanied by a concomitant memory loss. Tumor necrosis factors are a family of cytokines known to cause apoptosis. TNF-*α*, although once considered to be purely inflammatory, can also be produced in the brain under basal conditions. Signaling by TNF-*α* through the TNFR1 regulates synaptic strength via modulation of AMPA receptor expression [116]. TNF-*α* signaling can differentially affect adult hippocampal neurogenesis, with signaling through the TNFR1 a negative and TNFR2 a positive regulator [117]. TNF-*α* has an antineurogenic effect on NSCs and is upregulated in a number of neurodegenerative diseases [118]. Another example of a cytokine important for normal brain function is IL-1*β*, which is upregulated during LTP induction and maintenance [119]. IL1R1 is expressed by NSCs of the dentate gyrus but not the SVZ [120] and IL-1*β* decreases hippocampal NSC proliferation, via activation of NFkB signaling [121] and elevated levels of IL-1*β* are observed in the brains of patients suffering neurodegenerative diseases [122]. IL-1*β* administration impairs spatial learning and memory in the water maze [123], spatial active avoidance test [124], radial arm maze [125], and impaired hippocampus-dependent contextual fear conditioning [126]. IFN-*γ* is a proinflammatory cytokine that is able to exert both positive and negative effects on adult neurogenesis depending on the neurogenic niche. IFN-*γ* decreases NSC proliferation in the adult SVZ both* in vitro* and* in vivo* [127] but leads to an increase in neurogenesis in the dentate gyrus of adult mice and ameliorates spatial learning and memory performance [128].

### 5.2. Chemokines

Chemokines are a family of small, secreted cytokines that guide the migration of cells along a concentration gradient. Stromal cell-derived factor 1-alpha (SDF-1*α*; CXCL12) is released from activated astrocytes and signals NSCs to migrate to the site of neuronal damage [129, 130]. It also promotes NSC proliferation [129, 130] and survival [131] via its receptors CXCR4 and CXCR7, which are highly expressed on NSCs [132, 133]. Using CXCR4 depletion mice, it was shown that CXCR4 signaling, in addition to maintaining the NSC pool, also specified the inner third of the granule cell layer as the site of immature neuron differentiation [134]. Another chemokine that is upregulated in the brain following inflammation is monocyte chemotactic protein-1 (MCP-1; CCL2). TNF-*α* increases the expression of MCP-1, which induces NSC migration mediated via its receptor CCR2 that is highly expressed on NSCs [135–137].

### 5.3. Neurotransmitters

In addition to cytokine regulation, immune cells, most predominantly T cells, can produce and respond to common neurotransmitters such as acetylcholine, glutamate, dopamine, and serotonin [138–140]. These neurotransmitters are important for modulating learning, memory, and LTP, and they affect not only neurons but also the production and secretion of inflammatory factors from astrocytes and microglia [141]. Neurotransmitter cross talk can be bidirectional, with the CNS affecting the immune system and vice versa. Some antidepressants (including fluoxetine) act as immune modulators by shifting the T cells from a proinflammatory to an anti-inflammatory profile [139]. Microglia express functional dopamine receptors (D_1_ and D_2_) and their activation can decrease nitric oxide production after LPS stimulation [142, 143]. Systemically produced dopamine can transiently alleviate the suppression of autoimmune activity of Tregs allowing a neuroprotective response after injury [138]. Noradrenalin is important in maintaining brain homeostasis and noradrenalin depletion can contribute to the neuroinflammatory processes that lead to neurodegenerative diseases [144]. Noradrenalin can also change the cytokine production profile of T cells and activate hematopoietic stem cells to produce immune cells.

### 5.4. Major Histocompatibility Complex Class I

The MHC I functions to display fragments of non-self-proteins to cytotoxic T cells and thus is crucial for the initiation and regulation of adaptive immune responses. Until Corriveau et al. demonstrated neuronal expression in 1998 it was believed that neurons were one of the few cell types devoid of MHC I expression [145]. In the brain, it has been suggested that MHC I molecules have distinct neuronal functions that differ from their role in cellular immunity, such as the regulation of synaptic function and brain development [146, 147]. MHC I is expressed at the synapses [146] and neurons in MHC I KO mice have enhanced synaptic plasticity, increased excitability and higher frequency of miniature excitatory postsynaptic currents (mEPSPs), increased LTP, and decreased LTD [146, 148]. Furthermore, the ablation of MHC I resulted in improved behavioral recovery after stroke, indicating an enhancement of synaptic plasticity in the absence of MHC I [149]. A recent study suggests that MHC I might at least in part be involved in mediating the age-related decline in neurogenesis and cognitive function observed after exposure to an aged systemic environment, as no effects of aged blood were observed in young heterochronic parabionts with reduced MHC I cell surface expression [6, 7, 150]. The specific role of more than 50 MHC I molecules [147] and the dual role of MHC I in immune and neurogenic regulation remain unknown.

### 5.5. Toll-Like Receptors

Toll-like receptors (TLRs) are receptors expressed by sentinel cells at the body's first line of defense that recognize conserved molecules shared by pathogens. TLRs are also associated with the differentiation of stem cells, including hematopoietic and mesenchymal stem cells [151, 152]. Many members of the TLR family are expressed in the brain; however studies mainly focus on their role in pathological brain processes and their specific role in normal brain function is still not completely clear [153]. NSCs express TLR2 and TLR4 and in mice these receptors are involved in NSC proliferation and differentiation [154]. In rats, after TLR activation, NSCs expressing TLR2 and TLR4 produce proinflammatory cytokines, including TNF-*α*; however proliferation and differentiation are not affected [155].

## 6. Blood Modulates Brain Function

As we have shown above, the microvasculature brings circulating immune cells in close proximity to the hippocampal stem cell niche. These peripheral immune cells together with populations of brain resident immune cells secrete immune molecules and provide a common language that enables cross talk between the immune system and neurogenic niche. Aging represents one clear example of a situation in which the dramatic effect of neuroimmunological cross talk becomes apparent.

Adult hippocampal neurogenesis decreases with age, as shown by a strong decrease in proliferation, differentiation, and survival of newly born neurons, especially during the first few months of life in a rodent (or years in humans) [5, 156, 157]. Furthermore the capability to adapt to new experiences is dramatically decreased with age [158], although this statement has been questioned recently. Irrespective of this, the age-related impairment in adult neurogenesis and brain plasticity was linked to an altered systemic environment, with a parabiotic connection between the blood systems of aged and young mice reversing the aging effect in old animals [7, 8]. The systemic environment from young mice enhanced synaptic plasticity and cognition, as well as neurogenesis in the old parabionts, as determined by an increased number of proliferating BrdU-positive cells, Sox2-positive progenitor cells, and doublecortin-positive immature neurons [7]. Moreover, intravenous injections of plasma isolated from old mice into young animals showed the opposite effect, indicating that blood-borne factors mediate the decrease in adult neurogenesis during aging [7]. In a follow-up study from Villeda and colleagues, the administration of young plasma to aged mice rescued the age-related cognitive deficit with these mice displaying improvements in hippocampal-dependent learning and memory water maze tasks and contextual fear conditioning compared to aged mice receiving aged plasma [8]. Interestingly, they identified an increase in circulating immune-related factors, including the eotaxin chemokine C-C motif ligand 11 (CCL11), in the blood of aged mice and showed that this was associated with impaired cognitive function and decreased neurogenesis [7]. Of note, circulating CCL11 crosses the BBB via influx but also efflux transporters for CCL11, indicating an important role of the BBB in controlling CCL11 levels in the brain [159]. The altered carrier capacity of the BBB in aged individuals [27] indicates a possible link between the raised systemic levels of CCL11 and the correlating cognitive impairment in old mice [7]. CCL11 is one interesting example to highlight the connection between the aging brain and the immune system; however, it is clear that systemic changes are more complex and not limited to the effect of a single factor.

Further studies confirm the relationship between systemic changes that occur during aging and adult neurogenesis. As observed in the hippocampus, Katsimpardi et al. reported the effects of young systemic factors and their capacity to enhance neurogenesis in old heterochronic parabionts in the SVZ [6]. These studies highlight the crucial role of the systemic environment and the route via the blood stream in regulating adult neurogenesis. The precise mechanisms of how the immune system, following physiological stimuli including learning and physical activity, contributes to orchestrating changes in neurogenesis still remain largely unknown.

## 7. From Blood to Brain: Lessons from the Crayfish

Evidence is emerging that the nervous and circulatory systems are more tightly linked than thought. Although neural stem cell research has focused on mammals, primarily rodents and humans, stem cell niches are also found in the brains of crustaceans [160]. Within this niche a pool of dividing cells differentiate into mature olfactory interneurons. Surprisingly, these cells are not self-renewing stem cells but instead more restricted progenitors that travel from a distant neurogenic niche on the ventral surface of the brain. It was recently shown that blood cells are the most likely source of the neural progenitor cells [161]. Another example where neural stem cells are externally supplied is in the primitive invertebrates, flatworms [162]. This suggests that blood derived neural stem cells are an evolutionary ancient phenomenon but one that could provide an excellent model system to study the interaction between the peripheral immune system and neural stem cells.

## 8. Concluding Remarks

Neuroimmunological cross talk has been predominantly studied in disease. However, there is mounting evidence that immune cells are involved not only in neuroinflammation in response to injury or disease, but also in the maintenance of brain homeostasis under nonpathological conditions. Although we are making great headway in this direction, there are still many fundamental questions that remain to be answered. For example, what is the role of infiltrating peripheral immune cells, including B cells, natural killer cells, and dendritic cells in normal brain function? What are the exact mechanism and location of T cell entry into the brain? Once inside the niche, how do T cells interact with resident immune cells including astrocytes and microglia, and what are the exact consequences of these actions under physiological conditions? Are the same mechanisms utilized for immune cell activation in steady state and disease conditions—is it just a matter of balance? Can the quiescent neural stem cells be regulated/activated by immune signals? Are immune cells involved in regulating the increase in adult hippocampal neurogenesis following stimuli such as physical exercise and learning? By addressing these questions, a more complete picture of the role that the immune system plays in normal brain function can be obtained. In particular, it seems likely that some yet unexplained aspects of adult neurogenesis may in fact be under immune regulation. We have shown above that the fields of neuroscience and immunology do indeed share a common language, and a synthesis of the two will be a fruitful area of future research.
